# To do or not to do: Angiotensin converting enzyme inhibitors/angiotensin receptor blocker in COVID-19 elderly patients

**DOI:** 10.17179/excli2021-3821

**Published:** 2021-07-06

**Authors:** Naga Venkata Rama Krishna Vura, Rashi Sandooja, Amena Firoz

**Affiliations:** 1 Department of Internal Medicine, Cleveland Clinic Akron General, Akron, Ohio; 2 Pondicherry Institute of Medical Sciences, India

## ⁯

***Dear Editor,***

Severe acute respiratory syndrome coronavirus 2 (SARS-CoV-2), the causative organism of coronavirus disease-19 (COVID-19) is tied to the 2020 pandemic that originally began at the end of December 2019 in Wuhan, Hubei province, China. Epidemiological studies have shown that SARS-CoV-2 is known to cause increased mortality in adults beyond the sixth decade of life; similar findings were noticed consistently across the orb; the reason for the differential clinical severity remains murky. The higher prevalence of cardiovascular disease (CVD), hypertension, and diabetes mellitus in older adults infected with COVID-19 are linked to deteriorating the prognosis. The majority of the older adults are known to be taking either angiotensin-converting enzyme inhibitors/angiotensin receptor blockers (ACEl/ARBs) for various aforementioned causes. SARS-CoV-2 uses its viral spike glycoprotein ectodomain to bind the angiotensin-converting enzyme 2 (ACE2) receptor to gain entry into the human cells (Walls et al, 2020[15]). Preclinical studies have suggested that the renin-angiotensin-aldosterone system (RAAS) inhibitors may increase ACE2 expression, raising concerns regarding their safety in patients with COVID-19. This issue sparked a debate regarding the use of ACEl/ARBs because of the association between ACE2 and SARS-CoV-2. We review recent research to provide evidence-based recommendations amid Covid-19 in older adults using ACEI/ARBs. 

### Renin-Angiotensin System (RAS)

**Figure 1:**
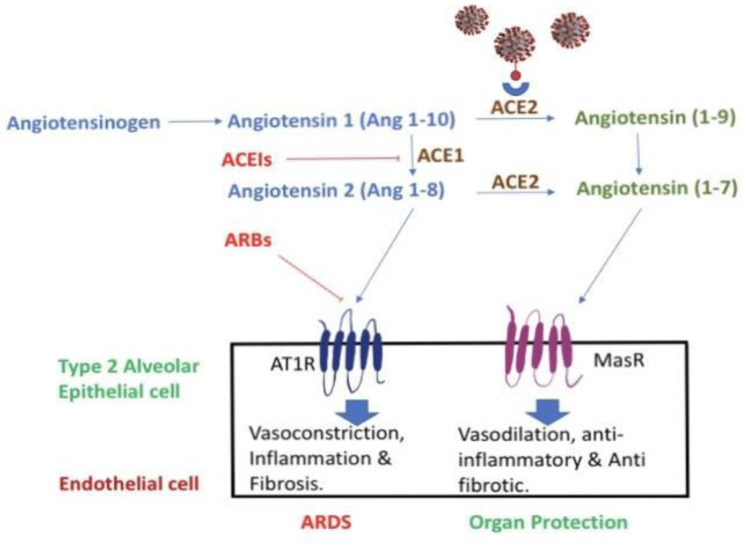
Schematic representation of the RAAS system and the effects of RAAS inhibitors in normal physiology and possible correlation with COVID-19 infection. ACE1 converts Angiotensin I to angiotensin II. Angiotensin II is responsible for acute lung injury and ACE2 metabolizes angiotensin II to angiotensin_1-7_. ACE II acts as the receptor for viral binding for SARS-CoV-2.

The initial rate-controlling step in RAS is renin. Renin is responsible for hydrolyzing angiotensinogen to angiotensin I. Angiotensin I is further converted to angiotensin II by angiotensin-converting enzyme (ACE1). Angiotensin II binds and activates angiotensin II receptor type 1, this activates downstream pro-inflammatory actions, including vasoconstriction and cell proliferation, responsible for acute lung injury seen in COVID-19 patients. ACE2 physiologically counters RAAS activation by metabolizing angiotensin II to angiotensin_1-7 _and angiotensin I to angiotensin_1-9. _Angiotensin_1-9 _binds to the Mas receptor which leads to anti-inflammatory and vasodilation. Both ACE1 and ACE2 are members of the ACE family of dipeptidyl carboxydipeptidases (Bavishi et al., 2020[2]). ACE2 is predominantly expressed in alveolar epithelium, bronchiolar epithelium, endothelium, smooth muscles of pulmonary vessels (Xie et al., 2006[17]). ACE2 exists in two forms: a structural transmembrane protein with an extracellular domain that serves as a receptor for spike protein of SARS-CoV-2 and a soluble form that represents the circulating ACE2. Observational studies have shown that urinary ACE2 levels were substantially increased in hypertensive and diabetic patients treated with ACEI/ARBs. A study by Liu et al. showed higher angiotensin II levels in patients infected with SARS-CoV-2 that was linearly associated with viral load and lung injury (Liu et al., 2020[9]). Based on these observations, one can contemplate the use of ACEI/ARBs can increase ACE2 expression and intensify SARS-CoV-2 entry leading to severe COVID-19 (Figure 1(Fig. 1)).

### Age-related changes

In an epidemiological analysis conducted in Wuhan in patients infected with COVID-19, it was observed that patients above the age of 59 years were 5.1 (4.2-6.1) times more likely to die after developing symptoms. Furthermore, the risk of symptomatic infection also increased with age (Wu et al., 2020[16]). In fact, a retrospective cohort study identified older age as the leading risk factor for mortality in patients infected with SARS-CoV-2 (Zhou et al., 2020[19]).

It is crucial to understand age-related alterations in ACE2 expression. Aging is associated with a decline in the ACE2 expression in the rat lungs (Xie et al., 2006[17]). In older adults with comorbidities such as hypertension and diabetes, ACE2 is downregulated as well. This decline in ACE2 levels is followed by decreased clearance of pro-inflammatory Angiotensin II, thus explaining the higher severity of disease seen in older individuals. Moreover, binding of SARS-CoV-2 with ACE2 for viral entry, further decreased the cell surface expression of the receptor, leading to exaggerated signaling by the Angiotensin II (AlGhatrif et al., 2020[1]).

Paradoxically decline in ACE2 expression in older individuals could significantly lower the incidence of disease in this subset of the population as ACE2 acts as the receptor for the SARS-Co-V-2 (AlGhatrif et al., 2020[1]). The number of cases in South Korea was the highest in the age group 20-29 years old. Interestingly, epidemiological data of SARS are the predominance of the young adult population. Cellular entry receptor is similar for both SARS-CoV-1 and SARS-CoV-2; this could be explained by reduced expression of ACE2 in older individuals (Xie et al., 2006[17]).

A large proportion of the elderly population are on medications including ACE inhibitors and ARB's. Animal studies in the past have shown that blockade of angiotensin II synthesis with ACE inhibitor or activity with ARBs is associated with an increase in ACE2 gene expression and activity (Ferrario et al., 2005[5]). Higher urinary ACE2 levels are seen in hypertensives treated with ARBs. This is the basis of the hypothesis that patients treated with ACEI/ARBs have augmented ACE2 activity leading to worse prognosis in COVID-19 patients. Conversely, it can be hypothesized that increased ACE2 activity would lead to increased degradation of Angiotensin II, and may prevent the deleterious effects of angiotensin II described above. 

There have been prior animal studies that have concluded that ACEI (Captopril) have had protective effects on oleic acid-induced acute lung injury in rats (He et al., 2007[6]). In 2017, Kim et al., in their retrospective study concluded that RAAS inhibitors may have protective effects in ARDS in humans as well (Kim et al., 2017[7]). In another meta-analysis of 37 studies - ACE inhibitors but not ARB's were associated with reduced risk of pneumonia (Caldeira et al., 2012[3]).

### Evidence

There is limited evidence available to date. A retrospective study done in China by Li et al. showed no association with disease severity in hypertensive patients taking ACEI/ARBs (Li et al., 2020[8]). Another retrospective cohort study done by Mehta et al. (2020[13]) concluded with no association between ACEI and ARB use and COVID-19 positivity.

Please refer to Table 1(Tab. 1) (References in Table 1: Cohen et al., 2021[4]; Lopes et al., 2021[10]; Mancia et al., 2020[11]; Mehra et al., 2020[12]; Mehta et al., 2020[13]; Reynolds et al., 2020[14]; Zhang et al., 2020[18]) for all the investigations that were completed to date and their conclusions.

### Conclusion

There is no sufficient evidence at this time to stop ACEI/ARBs in older adults. Most societies recommend continuing these medications when patients already on them. Moreover, they do not recommend initiating ACE inhibitors/ARBs in COVID-19 patients unless another clinical indication (like hypertension, diabetes). Based on available studies and society guidelines one should not hold ACEI/ARBs at this time. Much larger studies are needed for addressing this matter to provide evidence-based recommendations in the near future. We recommend continuation of renin-angiotensin system inhibitors safely in COVID-19 infected patients which are concordant with international society recommendations. 

## Conflict of interest

The authors declare no competing interests.

## Figures and Tables

**Table 1:**
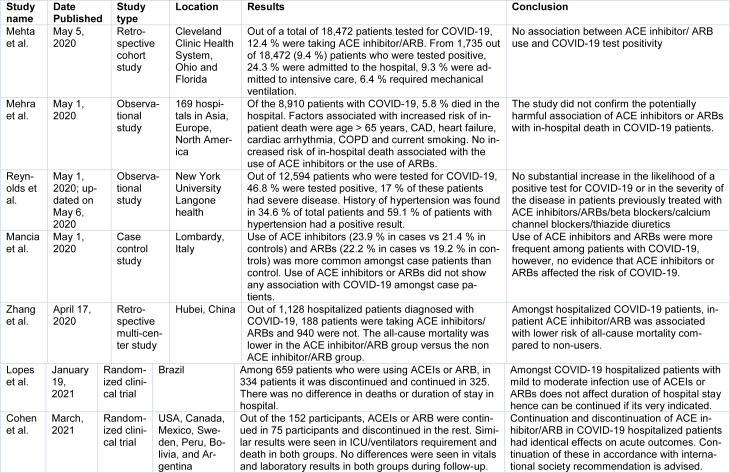
Studies that we reviewed about the use of ACEI/ARB in COVID patients
